# Barriers and challenges of safe traffic behavior in Jolfa City - a qualitative study 

**Published:** 2019-08

**Authors:** Morteza Haghighi, Yousef Dadashzadeh, Fatemeh Moniri, Mohammad Saadati, Mir Bahador Yazdani

**Affiliations:** ^*a*^Road Traffic Injury Research Center, Health Management and Safety Promotion Research Institute, Tabriz University of Medical Sciences, Tabriz, Iran.; ^*b*^Human Resources Management, Aras Free Zone.; ^*c*^Geography and Urban Planning , Department of Geography, Astara Branch, Islamic Azad university, Astara, Iran.

## Abstract

**Background:**

A large part of the traffic problems in many cities is related to the traffic behavior of citizens, ‎which was designed to explain traffic behaviors in the city of JOLFA and its upgrading ‎strategies. ‎

**Methods:**

This qualitative study was conducted in Jolfa. Gathering information through viewing and using ‎a video camera and shooting. Data analysis was carried out using conventional qualitative ‎content analysis method. ‎

**Results:**

According to the findings of the study, the barriers and challenges that affect traffic behavior in ‎the city of JOLFA can be summarized as three in general: Weakness in infrastructure / ‎environmental factors, Failure to implement traffic laws, Insecure behaviors of citizens And ‎travelers . ‎

**Conclusions:**

Considering the problems and solutions presented in this study based on observations, It can be ‎argued that the use of some programs, such as: providing educational programs, culture and ‎promoting safe traffic behavior among pedestrians and drivers, as well as reconstruction and ‎improvement of the necessary infrastructure, such as the construction of automated pedestrian ‎bridges, can, in addition to Improving traffic behavior prevented traffic accidents in the city of ‎JOLFA.‎

**Keywords:**

Traffic Attitudes, Jolfa City, Qualitative Study

